# MRI-compatible and sensorless haptic feedback for cable-driven medical robotics to perform teleoperated needle-based interventions

**DOI:** 10.1007/s11548-024-03267-z

**Published:** 2024-09-12

**Authors:** Ivan Vogt, Marcel Eisenmann, Anton Schlünz, Robert Kowal, Daniel Düx, Maximilian Thormann, Julian Glandorf, Seben Sena Yerdelen, Marilena Georgiades, Robert Odenbach, Bennet Hensen, Marcel Gutberlet, Frank Wacker, Frank Fischbach, Georg Rose

**Affiliations:** 1 Research Campus STIMULATE, Otto-von-Guericke University (OvGU), Magdeburg, Germany; 2 Faculty of Electrical Engineering and Information Technology, OvGU, Magdeburg, Germany; 3 Department of Radiology and Nuclear Medicine, University Hospital Magdeburg, Magdeburg, Germany; 4 Institute of Diagnostics and Interventional Radiology, Hannover Medical School, Hannover, Germany

**Keywords:** Sensorless force measurement, Teleoperation, Haptic feedback, Surgical robotics, Image-guided interventions, µRIGS

## Abstract

**Purpose:**

Surgical robotics have demonstrated their significance in assisting physicians during minimally invasive surgery. Especially, the integration of haptic and tactile feedback technologies can enhance the surgeon’s performance and overall patient outcomes. However, the current state-of-the-art lacks such interaction feedback opportunities, especially in robotic-assisted interventional magnetic resonance imaging (iMRI), which is gaining importance in clinical practice, specifically for percutaneous needle punctures.

**Methods:**

The cable-driven ‘Micropositioning Robotics for Image-Guided Surgery’ (µRIGS) system utilized the back-electromotive force effect of the stepper motor load to measure cable tensile forces without external sensors, employing the TMC5160 motor driver. The aim was to generate a sensorless haptic feedback (SHF) for remote needle advancement, incorporating collision detection and homing capabilities for internal automation processes. Three different phantoms capable of mimicking soft tissue were used to evaluate the difference in force feedback between manual needle puncture and the SHF, both technically and in terms of user experience.

**Results:**

The SHF achieved a sampling rate of 800 Hz and a mean force resolution of 0.26 ± 0.22 N, primarily dependent on motor current and rotation speed, with a mean maximum force of 15 N. In most cases, the SHF data aligned with the intended phantom-related force progression. The evaluation of the user study demonstrated no significant differences between the SHF technology and manual puncturing.

**Conclusion:**

The presented SHF of the µRIGS system introduced a novel MR-compatible technique to bridge the gap between medical robotics and interaction during real-time needle-based interventions.

## Introduction

Over the past two decades, there has been a significant paradigm shift in minimally invasive surgery, primarily attributed to the introduction and widespread adoption of robotic-assisted surgery (RAS). Apart from the well-known da Vinci system, there are more than 60 other RAS systems, among which 15 have obtained regulatory clearance for diverse application areas such as laparoscopy, neuro, spine, orthopedic, and vascular surgery [1]. RAS minimizes patient trauma, fostering faster recovery and improved user experiences. This results in shorter hospital stays, eases strain on critical units, and reduces physical stress on surgeons to enhance the overall hospital efficiency [2].

Present interfaces utilized in RAS exhibit an ergonomic console design, facilitating remote manipulation of instruments and visualization of the surgical site without any level of autonomy. To achieve the robotic status, current research and vision systems focus on areas such as data science, modeling, AI, and new sensors. Thus far, the principal modality for sensory feedback has been 3D visual observation facilitated by stereo endoscopes. However, the sense of touch, or haptics, has to date no established solutions in commercial RAS devices (Intuitive Surgical announced haptic feedback for Da Vinci 5 in 2025), despite its potential to optimize surgeon’s performance, enhance procedural success rates and time, and improve overall patient outcomes [3, 4].

In the current field of minimally invasive surgery, the importance of image-guided interventions is on the rise, particularly in interventional magnetic resonance imaging (iMRI), which offers no radiation exposure and best soft tissue contrast compared to interventional computer tomography (iCT) or interventional sonography (iUS) [5]. This trend paves the way for potential utilization of interventional robotics to enhance precision, accuracy, and safety, while also reducing radiation exposure, and enabling teleoperation of procedures. Although various robotic technologies are available in the state-of-the-art, only a limited number are commercially accessible, and none provide haptic or tactile feedback. In addition, the requirement of MRI safety limits the use of sensor methods. Current sensor technologies focus mainly on position detection, utilizing optical fibers, fiber Bragg gratings, piezoelectric, and pneumatic sensors. However, these technologies have drawbacks, such as size limitations, electromagnetic interference (EMI) issues, and high commercialization costs. Active sensor systems, especially when integrated into instruments, are relatively expensive, while their usage in mechanical components is constrained by size [6, 7].

Addressing the limitations of active sensors, a passive or sensorless method represents a further promising approach. Thereby, cable-driven robotics including motor torque measurements [8, 9] offer technical advantages, particularly for iMRI robotics, where ferromagnetic direct drives cannot be utilized due to their incompatibility with the MRI environment. This highlights the potential of the µRIGS system, utilizing non-metallic Bowden cables to ensure MRI compatibility including stepper motors for precise and remote-controlled positioning [10, 11]. In this work, a passive force measurement for µRIGS was developed, aiming to generate sensorless haptic feedback (SHF) for remote needle advancement using a force feedback controller, alongside collision detection and homing capabilities for internal automation processes. The evaluation focused on the performance of the force measurement and a user study to compare conventional needle puncturing with the SHF.

## Material and methods

**Fig. 1:**
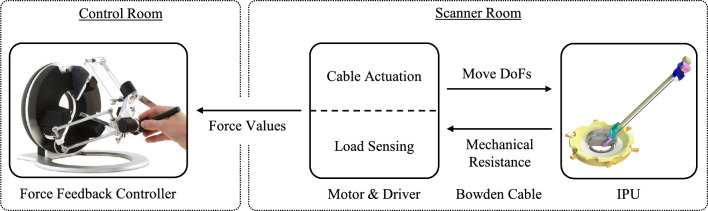
Overview of the SHF for the µRIGS system within the MRI suite

To establish a reliable SHF, further developments of the µRIGS device were performed (see Fig. 1). The instrument positioning unit (IPU) was optimized in stiffness, surface quality, and fit by using rigid resin (Heavy Duty, Formfutura BV) in a stereolithography printer.

Considering the drive unit (DU), 4-m-long Bowden cables transmitted the movement of stepper motors housed in an EMI-safe enclosure to the moving parts of the IPU’s respective degree of freedom (DoF) without interfering with the MRI. The Bowden cable consisted of a 0.9-mm-thick non-metallic high-performance pull cord (percent elongation at 20 N load: 0.1%) encased with a polytetrafluoroethylene (PTFE) liner to minimize friction on the polyethylene traction sleeve. Additionally, PTFE liner were added to different parts of the IPU to further reduce friction on the cord during deflections.

Those modifications provided less latency of motion transmission, less bending, and better sliding properties of the mechanical parts of each DoF without free play. The main focus of this work was to establish real-time haptic feedback while puncturing phantoms using a haptic controller (omega.6, Force Dimension) for user interaction. Additionally, the technology should provide sensorless force feedback for internal system collision detection and homing processes.

### Force sensing

The application of a load torque to the motor shaft induces changes in the electromechanical properties, facilitating a proportional adjustment of the mechanical forces acting on the Bowden cables. DC/servo motors exhibit limited torque availability at low speeds and encounter challenges with stand-still and direction change applications, whereas all the mentioned properties are required for µRIGS. Consequently, they incur higher costs and complexity compared to stepper motors when used in a hybrid mode for measuring torque loads. Therefore, NEMA 17 stepper motors with 0.46 Ncm (17HM19-2004S, StepperOnline) were implemented [12, 13].

The stepper motor driver TMC5160 offers the measurement of load torques using the back-electromotive force (EMF) constant and other drive settings, known as *StallGuard* [14]. The *Stall* represents the load angle (− 90° until + 90° depending on the rotation direction), which results in *st*-values with the possibility to update each full step. Beyond ± 90°or *st* = 0, the motor risks generating step losses. Therefore, the sampling frequency depends on the rotational speed of the motor *f*_rot_. Achieving an optimum trade-off of high *st* sampling frequency *f*_st_, sensitive *st* measurement, realistic needle infeed velocity *v*_F_ and sufficient but not excessively high torque to reliably and safely infeed the instrument, stepper motors with step angle of 0.9°, 10:1 planetary gearsets (EG17-G10, StepperOnline), and cable drums with a diameter of 22 mm were chosen.

To ensure an effective *st* measurement for the presented purpose, following motor driver parameters were set (see Table 1) with three *f*_rot_ and current *I*_M_ configurations at a supply voltage of 24 V in full step modus with a 256 microstep interpolation. This was done to analyze the performance for different needle feeding velocities.

**Table 1:** Setup of the motor driver to adjust individual moving and sensing performance

Individual settings		Resulting performance	
*f*_rot_ in rpm	*I*_M_ in mA	*f*_st_ in Hz	*v*_F_ in mm/s
60	250	400	7
90	280	600	11
120	280	800	15

### Haptic interaction

The omega.6 device is able to provide haptic feedback in 3 DoF. To simulate the needle infeed, 1 DoF was activated to allow movement of the omega.6 stylus in the upside-down direction. Before puncturing, 1000 *st*-values were acquired without any load to calculate an averaged zero value *st*_zero_. To use the highest possible force bandwidth of 12 N, the *st*-values were recalculated to transfer forces *f*_omega.6_ from 0 to 12 N in relation to *st*_zero_.

1 $$ f_{{{\text{omega}}.6}} = 12{\text{N}} \cdot \left( {{1 - }\frac{{{{st}}}}{{{{st}}_{{{\text{zero}}}} }}} \right) $$

In this work, omega.6 only transferred real-time forces to the user to maintain focus on the haptic feedback. The higher the applied force to the omega.6, the stronger the stylus is pushed in the upwards direction. The needle infeed was proceeded with a continuous feeding velocity of 10 mm/s.

### Phantom

**Fig. 2:**
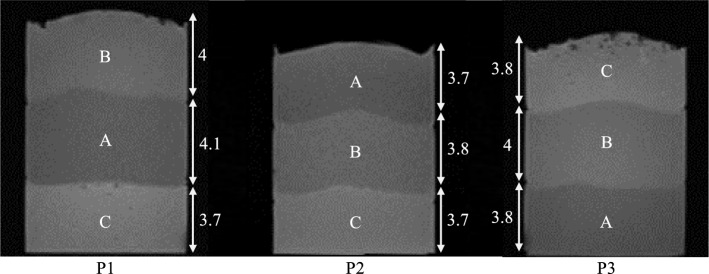
MRI of the phantoms (P1–P3) in transversal view. Image properties: T1-weighted VIBE sequence (TR = 3.5 ms, TE = 1.4 ms, and voxel size = 1 × 1 × 1.5 mm). Each layer is labeled with its height in mm. The brightness of each layer corresponds to its elastic properties, with brighter layers indicating higher stiffness

To evaluate the technology, different polyvinyl alcohol cryogel (PVA-C) phantoms were developed in accordance with [15] to mimic soft tissue for realistic needle punctures. Each cylindrical phantom (P1–P3, Ø 85 × 115 mm) represented a specific purpose, characterized by three-layer configurations. P1 simulated a soft area sandwiched between two harder layers, while P2 became progressively harder, and P3 became softer. The properties of the phantom layers are shown in the following Fig. 2 and Table 2.

**Table 2:** Composition (given in mass concentration m%), elastic property *E*, and soft tissue purpose of the respective phantom layer

Layer	PVA concentration in m%	*E* in kPa	Mimic soft tissue
A	10	4.6 ± 0.03	Liver, stomach, spleen, and intestine
B	15	25.8 ± 0.2	Muscles
C	23	83 ± 1.4	Kidney and tumors

The manufacturing process involved dissolving the PVA granules (KurarayPoval 15–99, Kuraray Europe GmbH) in distilled water while heating in a commercial microwave [16]. After one freeze–thaw cycle (FTC, freezing at − 20 °C for 9 h and thawing at 21 °C for 10 h), the PVA solution transformed into a cryogel. To prevent blending of different PVA layers, the first and second layers were frozen for 1 h each. Additionally, the 3D-printed phantom mold featured a form-fitting adapter to the µRIGS system, ensuring reproducibility.

### Experiments

The evaluation consisted of a calibration of *st*-values, phantom punctures in comparison with a compression testing machine (CTM, Xforce HP 50 N, zwickiLine Z0.5 TN, ZwickRoell GmbH) as reference with the SHF, and a user study to demonstrate the feasibility of the haptic behavior with the omega.6 device during needle infeed. All punctures were performed with a 16 G coaxial needle (KIM-16/15, Innovative Tomography Products GmbH). For the data postprocessing, MATLAB (The MathWorks Inc., version 24.1.0 (R2024a)) was used.

#### Force calibration

**Fig. 3:**
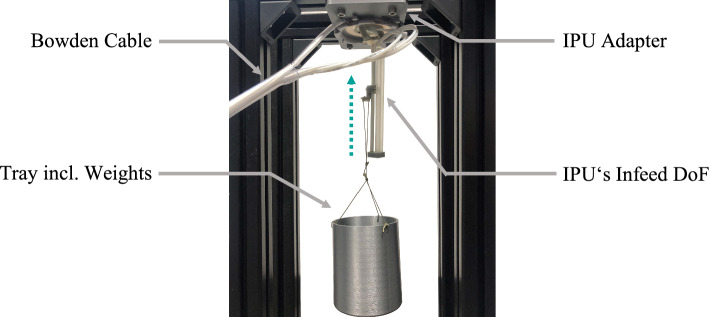
Force calibration setup. Various weights are pulled up by the infeed DoF against the force of gravity

To analyze the *st* related force *F*_s_, its theoretical resolution ∆*F*_s_ as well as *F*_max_, and the noise level at different kernel sizes *k* of a moving average (MA) filter, *st*-values were recorded using different calibrated weights. A tray including the weights (0 g, 100 g, 200 g, 400 g, 700 g, and 1000 g) was hung on the upside–down fixed IPU’s needle head holder to apply gravity forces in the needle infeed direction (see Fig. 3). The infeed movement was realized for each weight with parameters set as outlined in Table 1, covering a distance *s*_N_ of 5 cm.

#### Technical comparison punctures

**Fig. 4:**
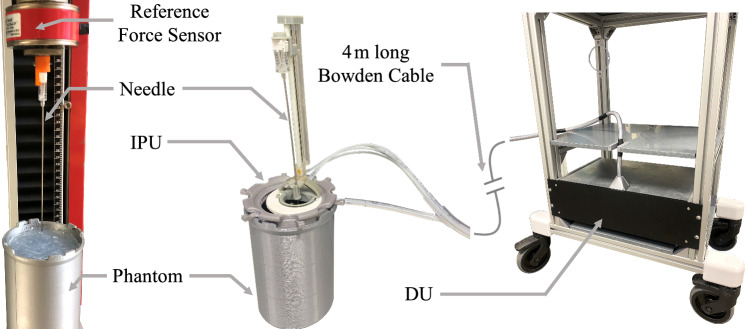
Puncturing setups to compare the µRIGS with a valid reference testing machine

To validate the technical effectiveness, the SHF was directly compared to the CTM while puncturing phantoms (see Fig. 4). Each phantom described in Sect. "Phantom" underwent five punctures in different areas and at different feeding velocities (see Table 1) using the CTM to determine reference data and phantom’s puncturing reproducibility. Once the homogeneity of each phantom layer was validated, each phantom was punctured with the SHF at the same feeding velocities as with the CTM. The calibration results of the MA filtering and *st–F*_s_ relation (see Sect. "Force calibration") were used to match SHF measurements to CTM data and conduct a Bland–Altman analysis for comparison.

#### User study

**Fig. 5:**
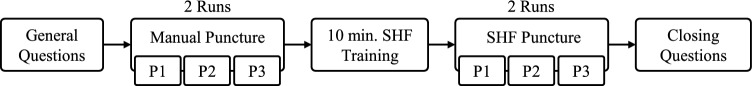
Procedural framework of the user study

The user study engaged five interventionists with different levels of professional experience and radiology focus fields (see Table 3). The procedural framework is outlined in Fig. 5. The needle insertion occurred under blind conditions, with the phantom concealed behind a visual barrier, while phantoms were selected randomly. During the initial phase of the user study, participants manually introduced the needle into the phantom (two randomized attempts), followed by 10 min of training with the SHF. In the second phase, participants used the SHF for insertion (two randomized attempts). This study design aimed to focus on participants’ ‘first impressions,’ preventing the development of muscle memory or guessing after repeated attempts.

**Table 3:** Information about the interventionists as participants of the user study

Participant	Interventional experience		
	Working years	Puncture quantity	Applications [Focus field] (Additional)
#1	0.5	10	[iMRI] biopsy/ablation therapy: liver (iCT) biopsy/drainage: kidney, abscess
#2	3	40	[iMRI] biopsy/ablation therapy: liver, prostate (iCT) drainage: abscess, kidney (iUS) biopsy: lymph node, muscle, breast, bone
#3	3	20	[iMRI] biopsy/ablation therapy: liver, kidney, muscle (iUS) biopsy: lymph node of axilla, neck, groin (iCT) biopsy/drainage: bone, abscess
#4	3.5	70	[iCT] biopsy/drainage: liver, kidney, lung, peritoneum (iMRI) brachy therapy: prostate
#5	2	40	[iUS] catheter/biopsy: vessels, lymph node (iCT) biopsy/drainage: liver, kidney, lung, abscess

Subsequent to each insertion, participants were prompted to specify the perceived number of layers, the order of each layer elasticity, and their degree of decision reliability in each assessment. The data of both methods for number of layers and layer order were compared using a Fisher’s exact test, while the degree of decision reliability was evaluated with a Wilcoxon test, both double sided at a significance level of 5%. The null hypothesis represented no difference between puncturing methods. Thereby, letter sequences indicate how many layers were recognized in the respective phantom and their arrangement. For example, ‘BAC’ means that three layers were recognized, with a medium–hard layer (B) at the beginning, a softer layer (A) in the middle, and a relatively hard layer (C) at the end. Three layers were assumed to be correct (1), while other quantities were considered incorrect (0). The correct layer order was determined based on predefined patterns for each phantom. For P1, a soft layer in the middle (e.g., BAC, CAB, and BCAD), for P2 increasingly tougher tissue (e.g., ABC, AB, and ABCD), and for P3, a soft layer at the end (e.g., CBA, BCA, and BA) was considered correct (1) while other combinations were incorrect (0). The degree of decision reliability of each puncture was analyzed using a 5-point Likert scale, where a higher number indicates a safer decision.

Upon completing the test sessions, inquiries were made concerning the general significance of haptic feedback during interventions, the realism of the phantom, as well as distinctions between manual and SHF advancements. During the user study, needle movements and participants’ voices were tracked with a camera to reproducibly evaluate user responses at each needle position.

## Results

### Force calibration

**Fig. 6:**
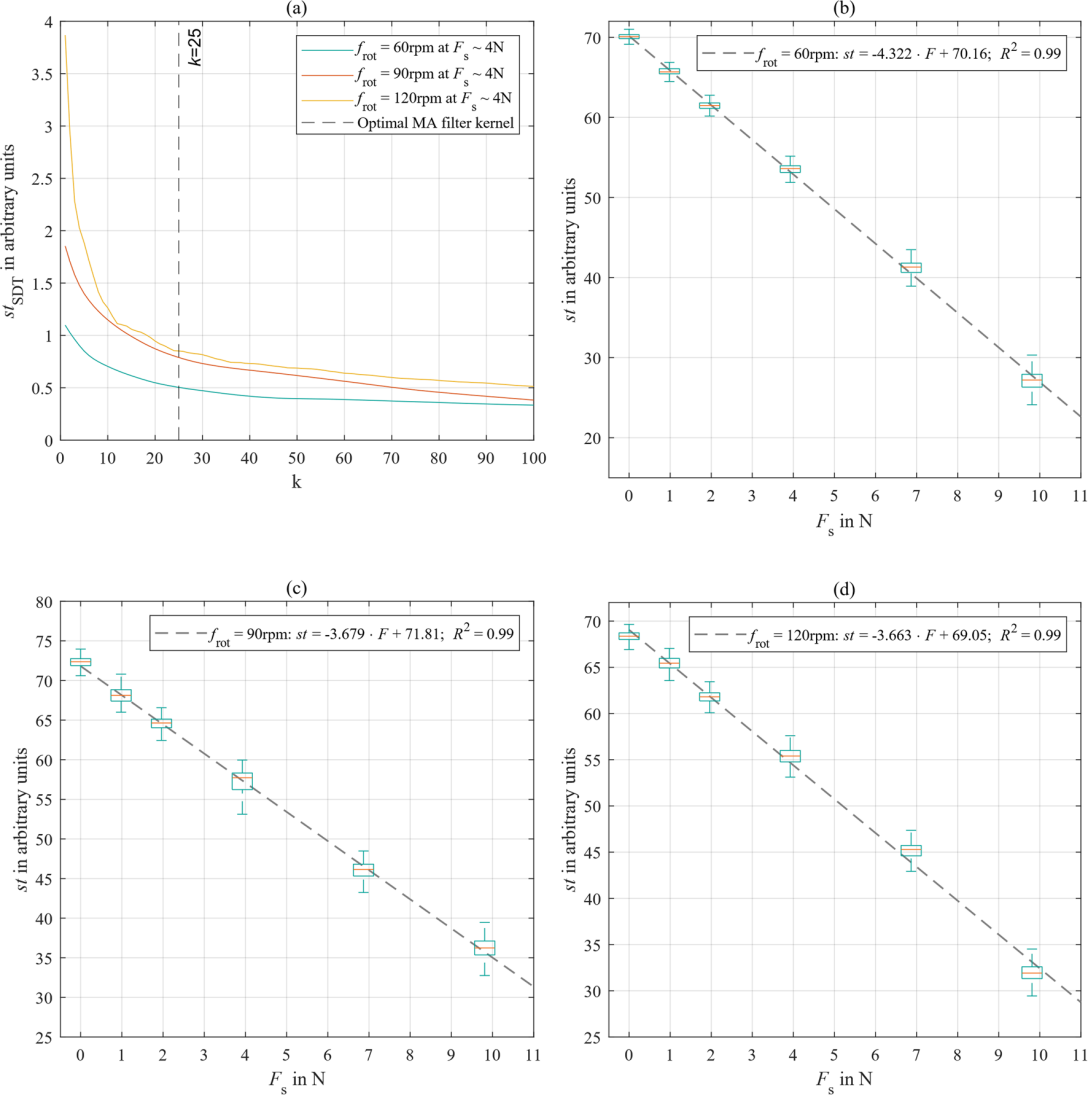
**a** Representative influence of the sliding kernel size *k* of a MA on the measurement inaccuracies of *st*-data at different *f*_rot_. **b–d** Boxplots representing the performance of *st* and its linear relation to *F*_s_ for each *f*_rot_

During interventional procedures, the SHF should utilize filtered values to ensure a smooth and real-time haptic experience with minimal latency. Since the raw *st*-data had a noise level, the influence of a MA filter with different sliding kernel sizes *k* on the standard deviation of *st*-values at different rotational speeds was analyzed (see Fig. 6a). In each case, the optimal balance between low latency and effective data smoothing was found for *k* = 25, reducing the noise level by over 40%. This filter size was then integrated into the following subsequent calibration, comparison analysis, and user experiments.

For each rotational speed and load conditions, boxplots illustrate the performance of filtered *st* and their linear relation to *F*_s_ (see Fig. 6b–d). This analysis yielded force resolutions with mean measurement uncertainty of ∆*F*_s60_ = 0.23 ± 0.16 N; ∆*F*_s90_ = 0.27 ± 0.3 N; and ∆*F*_s120_ = 0.27 ± 0.2 N. Generally, the noise of *st*-values increased with higher rotational speeds and mechanical loads. Considering that motor stall occurs when *st* = 0, linear regressions of the minimum values of raw *st*-data revealed maximum forces of *F*_max60_ = 14.5 N; *F*_max90_ = 16.5 N; and *F*_max120_ = 13.9 N.

### Technical comparison punctures

**Fig. 7:**
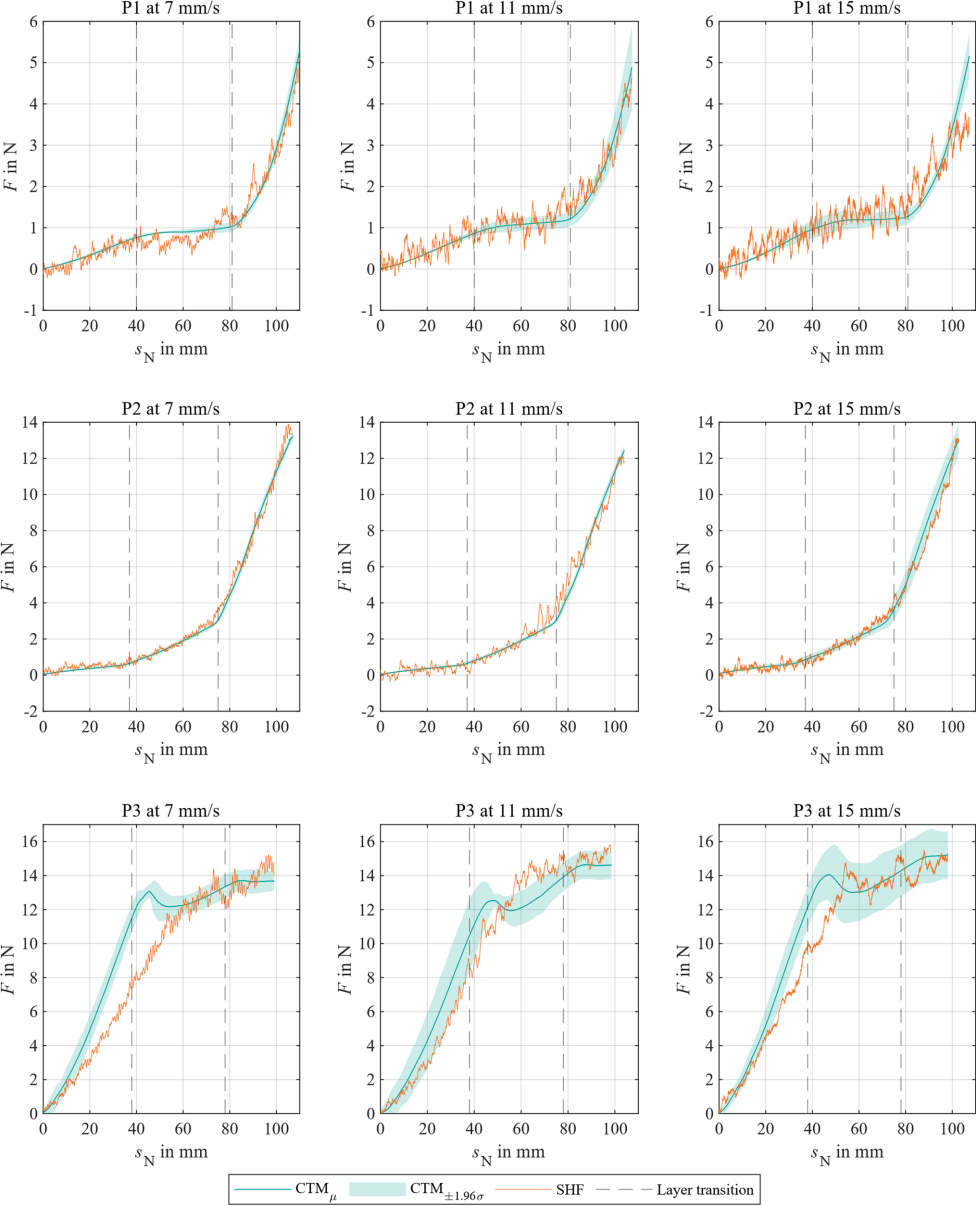
Comparison of needle forces of CTM with SHF during phantom (P1–P3) puncturing at different feeding velocities over feeding distance *s*_N_

The punctures with CTM resulted in reproducible force progressions for each phantom (see Fig. 7). Thereby, mean confidence intervals were determined: P1_±1.96 σ_ = 0.14 N, P2_±1.96 σ_ = 0.21 N, and P3_±1.96 σ_ = 1.05 N. Generally, the SHF data aligned with the intended force progression depending on the phantom, but it tended to exhibit a higher noise level with increased feeding velocities and mechanical loads.

**Fig. 8:**
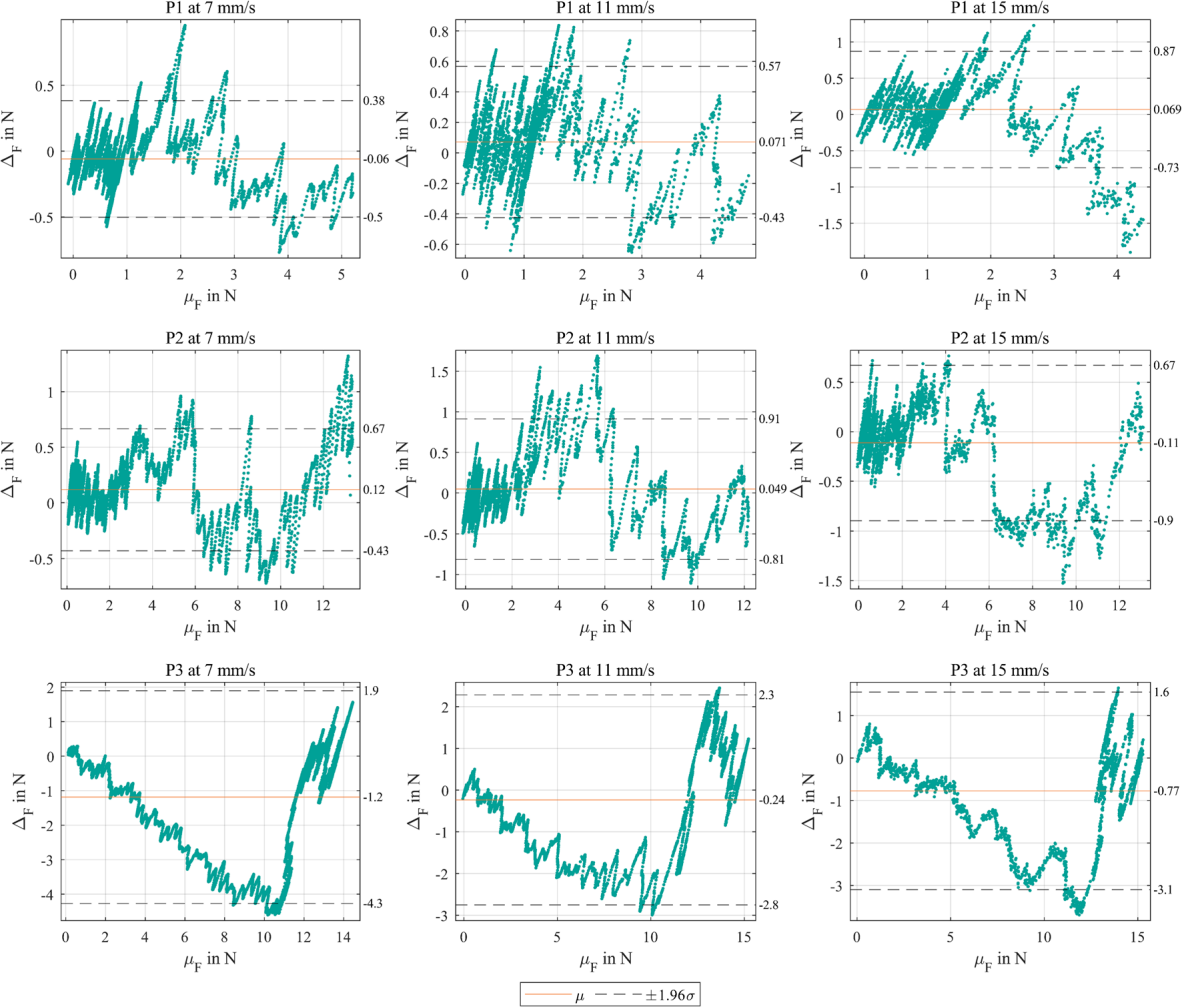
Bland–Altman analysis of the force difference ∆_F_ over mean force µ_F_ between CTM and SHF for each phantom (P1–P3) and feeding velocity

The Bland–Altman plots (see Fig. 8) present for P1 and P2 until ≈2 N the lowest ∆_F_ (± 0.5 N) in comparison with rising mechanical loads. As the µ_F_ increased, so did the ∆_F_. Thereby, SHF force curves could not follow the reference behavior with rising loads over time/distance. Consequently, ∆_F_ alternated between positive and negative values after each layer transition. The absolute force differences during the whole P1 and P2 puncturing µ_∆F_ were < 0.1 N on average. Notably, punctures in P3 exhibited highest ∆_F_ up to −4 N with µ_∆F_ = 0.72 N averaged across all velocities.

### User study

During the initial interrogation of the participants, it was discerned that haptic perception holds equivalent significance to imaging information when interpreting needle positioning. Both forms of feedback ensure redundant verification of needle positioning within tissue. Situational contingencies may elevate the importance of haptic perception; for instance, in cases of compromised image quality, liver tumors proximal to the diaphragm, or accurate biopsy extraction. Noteworthy elements for haptic sensation include fasciae, the diaphragm, liver/kidney capsules, bones, and abscesses. With increasing expertise, distinctions become perceptible in tissue depth, even with minor mechanical alterations. The velocity of needle advancement varies individually based on technique and experience. A common approach is to reach the fascia with higher velocities than regions that contain critical structures (such as large vessels, pleura, etc.) or those close to the target area.

Table 4 represents the puncturing experience of each participant using both methods in comparison with the intended phantom properties (see Fig. 2).

**Table 4:** Manual and with the SHF executed puncturing experience of each participant (#1–#5)

Method_Attempt	#1			#2			#3			#4			#5		
	P1	P2	P3	P1	P2	P3	P1	P2	P3	P1	P2	P3	P1	P2	P3
Phantom properties	BAC	ABC	CBA	BAC	ABC	CBA	BAC	ABC	CBA	BAC	ABC	CBA	BAC	ABC	CBA
Manual_1	AB	ABC	BAC	BAC	ABC	CBA	CAB	BAC	CBA	BAC	ABC	CBA	AB	ABC	BCA
Manual_2	BAC	AB	BAC	BAC	ABC	CBA	BCAD	ABCD	BCA	BAC	ABC	CAB	AB	ABC	BCA
SHF_1	BAC	ABC	BCA	ABC	ABC	CBA	ABC	CABD	ACB	AB	ABC	BAC	AB	ABC	ABC
SHF_2	ACB	ABC	ABC	ABC	AB	BCA	BAC	ABC	ABC	BAC	AB	BA	ABC	AB	ABC

Each phantom (P1–P3) is described in different layer orders (A–D$\hat{=}$soft–hard)

The quantitative evaluation of the study (see Table 5) involved the statistical comparison of the correctness of the perceived layer quantity and layer order considering their degree of decision reliability between both methods.

**Table 5:** User study evaluation with each participant (#1–#5) punctured each phantom (P1–P3) using each method

Metrics	Method_Attempt	#1			#2			#3			#4			#5			*p*-value
		P1	P2	P3	P1	P2	P3	P1	P2	P3	P1	P2	P3	P1	P2	P3	
Layer quantity	Manual_1	0	1	1	1	1	1	1	1	1	1	1	1	0	1	1	1
	Manual_2	1	0	1	1	1	1	0	0	1	1	1	1	0	1	1	
	SHF_1	1	1	1	1	1	1	1	0	1	0	1	1	0	1	1	
	SHF_2	1	1	1	1	0	1	1	1	1	1	0	0	1	0	1	
Layer order	Manual_1	0	1	0	1	1	1	1	0	1	1	1	1	0	1	1	0.17
	Manual_2	1	1	0	1	1	1	1	1	1	1	1	0	0	1	1	
	SHF_1	1	1	1	0	1	1	0	0	1	0	1	0	0	1	0	
	SHF_2	0	1	0	0	1	1	1	1	0	1	1	1	0	1	0	
Degree of decision reliability	Manual_1	4	3	3	4	4	3	4	3	4	4	3	3	3	5	3	0.31
	Manual_2	3	4	4	5	5	4	3	4	4	4	3	3	3	4	4	
	SHF_1	2	4	2	3	4	3	5	4	2	2	4	3	3	4	4	
	SHF_2	3	3	4	5	3	3	4	5	3	3	4	3	4	5	3	

The correctness (1: correct and 0: incorrect) of the perceived layer quantity and layer order was measured considering their degree of decision reliability (1: unsafe and 5: safe). The results of the three metrics provided the basis for statistical tests (*p*-values) to compare manual with SHF puncturing

The interpretation of the layer quantity was missed by ± 1 in ≈17% of cases, and the layer order was interpreted correctly in ≈70% in comparison with the intended phantom properties, regardless of the feeding method. In general, the manual puncturing performance had no significant differences to the SHF when interpreting the layer quantity (*p* = 1) and the layer order (*p* = 0.17). P2 was the most reliable phantom for the participants to interpret, being recognized correctly in ≈90% of the cases, with the highest equality of the puncturing method. The highest interpretation errors of the layer order with the SHF occurred during puncturing P1, where the soft layer between two harder layers was often not recognized. The overall degree of decision reliability had no significant difference between the manual feeding and the SHF method. Participants were able to rely on the SHF method to interpret the phantoms, especially after the first run, even with minimal prior training. The lowest degree of decision reliability was determined for P3 using SHF.

During the closing interrogation, phantom layers were declared to be realistic in terms of mechanical properties and valid for this type of user study. In a real setting, only the ratio of layer heights would change (e.g., 70–80% A-layer and the remainder B/C-layer). Despite the training lasting only 10 min, it was easy for the participants to handle the SHF and concentrate on haptic feedback without being able to influence or see the speed of the needle. Nevertheless, they would have benefited from a visual reference to the needle’s feed speed.

## Discussion

While the SHF setup maintained constant motor velocities and confirmed the linear relation between *st* and *F*_s_, introducing motor accelerations can disturb the accurate measurement of *st* and consequently affect the *F*_s_ behavior, especially for *f*_rot_ < 50 rpm [14]. This limits the technical implementation of a controller that simultaneously provides haptic feedback and actively adjusts the needle position in real time.

The CMT data indicate homogeneous phantom layers. However, due to the uneven layer heights across the puncturing area (see Fig. 2), force offsets during layer transitions were recognized, especially for P3, which had a C-layer at the beginning containing the most manufacturing-related air bubbles. This can lead to random puncturing errors affecting the comparison results.

According to Kern et al. [17], *f*_st_ should be at least 1 kHz in order to be able to reliably detect highly dynamic haptic events, which can be achieved with the SHF by setting higher *f*_rot_ but generating higher signal noise. Since the finger tips are capable to resolve forces in a < 1 mN range, the SHF provided a significantly lower sensitivity with a measurement uncertainty of ≈100% resulting from the limitations of the motor-based sensor method. Due to the Bowden cable elongation (2 mm at ≈15 N), the force curve could not follow the reference behavior with rising loads over time/distance. During the user study, this sensitivity loss was noticed by all participants. But as the participants noted that equal performance is not absolutely necessary if the MR/CT/US images could provide all relevant details, while the SHF offers gross support and redundant verification of the needle position.

The reached *F*_max_ ensures sufficient needle feeding force for puncturing soft tissue [18], while simultaneously protecting against system errors that could lead to incorrect feed positioning and thus tissue damage.

Participants with greater experience (#2, #4) found phantom interpretation to be the least challenging, relying more situationally on haptic sensation than on MR/CT/US images during interventions, indicating a sensitive perception and interpretation of haptics. Both manually and with the SHF, the most challenging event was the transition from harder to softer layers resulting in lowest recognition of the correct layer quantity and layer order, especially with SHF. Initially, higher needle frictional forces compared to further progression complicates the interpretation of a softer structure, as the required feed forces do not decrease absolutely, but either stagnate or progress with a flatter force gradient.

However, the small sample size of five participants in this user study limits the power of the quantitative evaluation as the results are not representative to the overall user population and may not achieve statistical significance. As Sauro and Lewis [19] emphasize that smaller sample sizes (5–10) are often sufficient to gain initial insights and identify 85% usability issues, the results of the presented pilot study confirm fundamental trends. For more comprehensive and generalizable results, a larger sample size (20–40) is required to ensure the statistical significance and accuracy of the metrics [19].

For future studies, participants expressed a preference for phantoms that could explicitly simulate fascia, liver/kidney capsule, or an abscess wall, as these tissue types are particularly sensitive to the touch and serve as orientation in the puncture process. Additionally, a higher significance level with the SHF can be achieved by incorporation with more phantom variations, > 30 participants with different interventional application fields and experience levels, conducting tests with and without imaging assistance, and involving active real-time needle positioning while receiving haptic feedback.

## Conclusion

This work presents a sensorless and real-time haptic feedback technology (SHF) using mechanical load changes of stepper motors to drive Bowden cables, maintaining MR safety and the compact design of the µRIGS system. In most cases, the SHF data aligned with the intended phantom-related force progression. During calibration processes, feedback data aided in collision detection and homing processes of moving parts of µRIGS. The evaluation of the user study revealed no significant differences in haptic experience between manual puncturing and SHF. Nevertheless, technical comparison analyses and qualitative results from participant interviews suggested sensitivity losses and haptic delays. Since the MR/CT/US images are the main feedback source for needle positioning, highly sensitive haptic feedback is not mandatory if sufficient imaging information can be provided. Thereby, the SHF offers gross haptically support and redundant verification of the needle position. This technology enables teleoperative interventions, which particularly can also benefit iCT by avoiding radiation exposure to the interventional team.

Future research and development will focus on mechanical optimizations to further minimize latency of cable drive units, as well as on real-time filters for linear prediction problems to enhance the haptic sensitivity. In a larger user study, the relevance of the presented interaction technology could be demonstrated for novel robotic-assisted and needle-based interventional workflows.
